# Supplementary material for: Virus-induced transposable element expression up-regulation in human and mouse host cells
Source: Life Sci Alliance. 2020 Jan 21;3(2):e201900536. doi: 10.26508/lsa.201900536 (PMC6977392; doi:10.26508/lsa.201900536)
Supplement: Supplementary file 8 [file LSA-2019-00536_TableS8.docx]

| Source | # upregulated TEs | % upregulated TEs |
| --- | --- | --- |
| Readthrough | 35 | 39.8% |
| Intron retention (IR) | 17 | 19.3% |
| Intergenic | 8 | 9.1% |
| IR through readthrough | 6 | 6.8% |
| IR or readthrough | 17 | 19.3% |
| Intergenic or readthrough | 5 | 5.7% |
| Total | 88 | 100% |

Table S8. Table summarizing transcriptional origins of top 88 shared DE TE (ERV and LINE) loci in the human genome that have been manually annotated. Annotations are in Supplemental Table 6.
